# Development and validation of the Informal Supporter Readiness Inventory (ISRI)

**DOI:** 10.1371/journal.pone.0296770

**Published:** 2024-03-11

**Authors:** Ryan L. Davies, Kylie Rice, Adam J. Rock

**Affiliations:** School of Psychology, University of New England, Armidale, New South Wales, Australia; Tabriz University of Medical Sciences, ISLAMIC REPUBLIC OF IRAN

## Abstract

**Objective:**

This article outlines the development and validation of the Informal Supporter Readiness Inventory (ISRI), based on the model developed by the present authors in (Davies, 2023). This scale assesses the readiness of informal supporters to intervene or provide support in situations of intimate partner violence (IPV).

**Methods:**

The research followed a three-phased procedure of item development, scale development, and scale evaluation; adhering to best practice guidelines for psychometric development and validation. This process provided empirical substantiation for the domains of the Model of Informal Supporter Readiness (Davies, 2023).

**Results:**

The 57-item ISRI incorporates four primary factors: normative, individual, goodman-emotional, and situational-assessment. These factors demonstrated robust internal consistency and factor structures. Additionally, the ISRI evidenced strong test-retest reliability, and both convergent and divergent validity. Although aligning closely with the Model of Informal Supporter Readiness, the scale revealed a nuanced bifurcation of situational factors into situational-emotional and situational-assessment.

**Discussion:**

The ISRI offers an important advancement in IPV research by highlighting the multifaceted nature of informal supporter intervention. The findings have several implications, from tailoring individualised supportive interventions to strengthening support networks and empowering survivors. The present study’s findings underscore the potential of adopting a social network-oriented approach to interventions in IPV scenarios. Applications for research and practice are discussed.

## Introduction

Intimate partner violence (IPV) against women is a significant public health issue globally. IPV affects almost one third (27%) of women worldwide [1] and has economic costs of $594 billion dollars per year in the United States alone [2]. IPV includes acts of physical, sexual, and psychological harm which are underpinned by the use of coercive control [3], and can have profound and lifelong consequences for survivors. In their study, Alsaker et al. [4] found that women subjected to IPV had a worse quality of life outcomes across eight key domains: physical functioning, social connections, routine activities, bodily pain, general mental health, emotional problems, vitality, and overall health perceptions. Potter et al. [5] found that both physical and mental health outcomes deteriorated further when multiple types of abuse were involved, a scenario more prevalent than IPV incidents involving a single type of abuse.

### IPV disclosure

Despite the significant consequences of IPV, survivors seldom report the abuse to formal supports (e.g., police, legal supports, health care professionals). It is estimated that only 6%-28% of survivors formally report their experiences to professional services [6, 7]. Survivors have reported significant barriers to reporting the violence, including fear of retaliation by the perpetrator [2], direct interference from the perpetrator in reporting attempts [8], fear of judgment from the professional [9], and a lack of trust in the professional [10]. Further exacerbating this issue, Hamberger et al. [11] found that healthcare workers frequently fail to systematically screen for IPV. This omission is often attributed to time constraints, absence of a clear policy or protocol, and conflicting care philosophies, which collectively diminish the opportunities for survivors to make a meaningful disclosure. Although there are barriers to disclosing IPV to the survivor’s social network, such as fear of negative reactions or causing familial shame [6], the majority of IPV disclosures are nevertheless made to family members or friends [12].

### Informal supporters

Social network members typically have greater availability and flexibility to support IPV survivors compared to professional supports [13]. However, the nature of support extended following disclosure can vary significantly, ranging from positive to negative. Survivors have identified several negative responses to disclosure as particularly unhelpful. These include directive suggestions or pressure to respond in specific ways, such as separating from the perpetrator [14], minimising the severity of the violence [15], blaming the victim for the violence [16], and future avoidance of the survivor [17, 18]. Conversely, survivors have reported positive forms of support as most helpful, particularly emotional support. In their review of the literature, Sylaska and Edwards [14] found that survivors most commonly reported emotional support from informal supporters, identifying empathetic listening and validation of their experiences, as the most helpful support type. Emotional support was followed by instrumental assistance [14], which refers to tangible help such as providing accommodation, childcare, and assistance with daily tasks [19, 20]. The final category of positive support identified by survivors was informational support, which includes the provision of advice and guidance on seeking appropriate professional support and interventions, such as counselling or reporting to the police [20].

### The benefits of informal supporters

Positive responses and assistance from informal supporters can have profound value for IPV survivors. Notably, social support can enhance the ongoing physical safety of survivors. According to Goodman et al. [21], women with higher levels of social support had a 20% probability of experiencing re-abuse within a 12-month period, compared to a 65% probability among women with lower levels of social support. Moreover, the long-term risk of abuse diminishes when social network members help survivors access professional resources [22]. Additionally, strong social networks support the emotional well-being and psychological health of survivors. Greater levels of social support have been associated with reductions in risk of suicide [23], feelings of self-blame [24], depressive symptoms [25], and posttraumatic stress symptoms [26]. Furthermore, numerous studies have found that social support is positively correlated with resilience [27], emotional stability [28], and improved quality of life outcomes [29]. Therefore, fostering supportive social networks is instrumental in mitigating the effects of IPV and promoting survivor recovery.

### Move towards a network-orientated approach

Given the significant role that strong social networks play in the wellbeing and safety of IPV survivors, there is increased focus on integrating formal and informal support services. Recently, researchers have identified a need to shift the focus of formal IPV interventions towards a social network-oriented approach [13, 30]. This approach suggests that professional service providers can assist IPV survivors to enhance safety by helping them strengthen their social networks, and supporting them to engage social network members in safety planning [30–33]. While some practitioners have shown openness to adopting a social network-oriented intervention approach [30, 33], and many are already incorporating some network-oriented practices into their work, many mainstream IPV service models have yet to adopt this integration as a core part of their support strategies [30, 34]. Therefore, challenges persist in implementing social network-oriented interventions. Among these challenges is the need for additional theory-based training and guidance regarding the process [30]. Further, advocates have highlighted the current difficulty in their ability to support survivors to distinguish between helpful and unhelpful social supports [13], making it challenging to determine where to direct encouragement effectively.

### Limitations of current measures of informal supporter suitability

Despite its importance, the task of identifying positive social supports to assist in survivor advocacy and support situations has yet to be comprehensively addressed from an empirical standpoint. The primary limitations of existing measures, aimed at determining an individual’s likelihood of offering a positive response to IPV survivors, is their grounding in the bystander model which emphasises the concept of *‘willingness’*, which primarily concerns an individual’s behavioural intention [35]. These models, while informative in some contexts, have several notable limitations when applied to IPV. For instance, they were not informed by the complex and layered relationships that social network members can have with survivors and perpetrators [36]. Their focus remains largely on the individual’s willingness to intervene, in the short term, while often overlooking the broader social norms and influences on a person’s action or inaction. Additionally, these models concentrate on immediate, one-time interventions and do not account for the need for long-term support strategies for IPV survivors [37]. This perspective provides limited utility to survivors and survivor safety advocates during the safety planning process, as a social network member who has agreed to be part of the safety plan has already demonstrated *‘willingness’*. Simply re-assessing their willingness adds little value. In contrast, *‘readiness’*, a concept that considers an individual’s motivation, capacity, and efficacy to intervene or provide support, holds greater utility [36]. *‘Readiness’* goes beyond the immediate intention and looks at the preparedness and ability of an individual to provide meaningful, ongoing support. This shift in focus, from *‘willingness’* to *‘readiness’*, could allow for a more comprehensive understanding of the potential role and capacity of each network member in the support process. Evaluating *‘readiness’* thus provides more actionable insights for developing effective and enduring safety plans for IPV survivors.

### The model of informal supporter readiness

This lack of unique theoretical conceptualisation of help-giving by social networks of IPV survivors was highlighted in a recent systematic review of informal supporters, prompting the development of the Model of Informal Supporter Readiness (MISR; [36]), which offers a more precise approach for conceptualising and assessing help-giving readiness. Based on current literature, published over the past 15 years, the model consists of three factors that are associated with increased help-giving readiness: 1) the normative factor, which considers the nature of help-giving social norms within the individual’s social network; 2) the individual factor, which considers the person’s beliefs about IPV and help-giving; and 3) the situational factor, which considers the unique interplay of relationships between the informal supporter, the survivor, and the perpetrator.

### Aims

Based on MISR [36], the current study proposed the development of the Informal Supporter Readiness Inventory (ISRI). This tool aims to address existing limitations in measuring the readiness of social network members to support IPV survivors. Importantly, the ISRI moves beyond the concept of ‘*willingness’–*which typically pertains to mere behavioural intention–and introduces *‘readiness’* as a more practical and actionable measure. This shift acknowledges that readiness for help-giving is not solely about intention; it is also informed by motivation, efficacy, prevailing social norms, and the specific dynamics of each interpersonal relationship. To ensure the robustness and utility of the ISRI, our study had several key objectives.

1. we aimed to validate the factor structure of the ISRI through confirmatory factor analysis
2. we planned to assess the ISRI’s reliability, by examining both its internal consistency and stability over time (test-retest reliability)
3. we intended to evaluate the ISRI’s convergent and divergent validity, to ensure that it is accurately capturing the construct of help-giving readiness, while being distinct from other constructs
4. Through Receiver Operator Characteristic Analysis, we aimed to determine the predictive validity of the ISRI and establish clinical cut-off scores.

## Method

This study followed the three-phased process for scale development outlined by Boateng et al. [38]. This process, as detailed below, consists of item development (phase 1), scale development (phase 2), and lastly, an evaluation of the scale’s validity and reliability (phase 3).

### Item development

#### Domain identification

The construct of informal supporter readiness was operationalised in the MISR, which was developed from the findings of a systematic review of factors influencing the likelihood of informal supporter intervention or assistance to survivors of IPV [36]. Boateng et al. [38] stated that when developing scales from a framework or model, the domains should be determined *a priori*, and each domain should be clearly defined. The MISR [36] includes three defined components, which are detailed below.

The normative factor [36] focuses on the nature of help-giving social norms within an individual’s social network and comprises three subfactors. Firstly, *injunctive norms* guide individual behaviour by setting perceptions of what actions are typically approved or disapproved. These norms define socially acceptable and unacceptable behaviours, and help an individual determine whether intervening in the context of IPV would be seen as a positive act or an inappropriate intrusion into a private matter. Second, *descriptive norms* relate to the actual observed behaviours of significant others. Rather than reflecting beliefs about what should be done, descriptive norms are about what is actually done; they give insight into the typical behaviour within the social network. For instance, if members of one’s social network often assist IPV survivors, this is likely to influence the individual’s perception of such actions as being appropriate. Lastly, an individual’s *sense of community and belonging* is associated with their motivation to comply with these social norms. This implies that one’s connection to their community can influence the extent to which they align their actions with these norms.

The individual factor [36] considers an individual’s beliefs about IPV and help-giving, and contains four sub-factors. First, *self-efficacy* represents the informal supporter’s confidence in their ability to provide effective help, as well as their belief in possessing the necessary skills and knowledge to do so. Second, the *acceptability of IPV*, evaluates an individual’s belief in the appropriateness of IPV within relationships. A higher level of acceptability may be associated with victim-blaming attitudes, suggesting that survivors may be at fault for the violence (even partially), and thus are undeserving of help. Third, the *social responsibility* sub-factor reflects an individual’s belief in their broader responsibility to respond to IPV. Finally, the *experience of violence* subfactor seeks to comprehend the impact that witnessing or experiencing IPV has on an individual’s self-perception of their ability to be an effective informal supporter.

The Situational Factor [36] considers the unique dynamics among the informal supporter, the survivor, and the perpetrator. This factor is broken down into nine sub-factors. First, two *Relationship* subfactors measure the level of closeness the informal supporter shares with both the survivor and the perpetrator. It is likely that individuals are more inclined to expend personal resources to assist those with whom they share a stronger bond. Second, the *Abuse* sub-factor assesses the severity, frequency, and chronicity of the abuse. This evaluation allows the informal supporter to gauge the overall impact of the abuse on the survivor, thereby informing the level of response required. Third, two *Responsibility* subfactors evaluate the informal supporter’s perception of who is more accountable for the abuse–the perpetrator or the survivor. A clearer attribution of the violence to the perpetrator may lead to greater intentions to provide help. Fourth, *Empathy* gauges the informal supporter’s level of sympathetic concern for the survivor. An emotional connection with the survivor may be related to a sense of moral obligation in the supporter to extend help. Fifth, the *Risk* sub-factor measures the informal supporter’s perception of the level of risk (to the survivor, themselves, or others) should they provide help. Higher levels of perceived risk may diminish the intention to help, as the potential negative outcomes might be considered to outweigh the benefits of providing support. Sixth, *Change Readiness* evaluates the informal supporter’s belief in the survivor’s preparedness to receive support. Supporters may be less likely to offer help if they perceive that the survivor is not ready or that the assistance may be unhelpful. Lastly, the *Emotional Response* sub-factor assesses the level of emotional distress experienced by the informal supporter upon recognising instances of IPV. High levels of distress may overwhelm supporters, inhibiting their ability to provide support.

#### Item generation

The initial item pool was generated using both inductive and deductive methods, as suggested by Morgado et al. [39]. Items were developed deductively from the conceptual definitions of sub-factors as defined above, as well as through reviewing existing measures of the sub-factors. The inductive item generation stage took place during evaluations by both experts and the target population (i.e., informal supporters of IPV survivors), in which additional items could be proposed. The individual items were crafted and refined multiple times by the authors (RD in consultation with KR and AR). Meetings were conducted to achieve consensus on the items, with the process continuing until the items were deemed by the authors to be representative of the conceptual definitions of the subfactors of the ISRI [40]. An initial pool of 76 items, twice the number anticipated for the final scale based on the number of subfactors, was agreed upon to allow for subsequent item reduction [40, 41]. Given that responses were to be measured using a bipolar scale, to capture both the negative and positive aspects of the constructs, a decision was made to score items on a seven-point Likert-type scale to enhance reliability [38, 42].

#### Content validity

In accordance with best practice, five expert judges were enlisted to review the initial item pool [38]. These experts were practicing psychologists with research or clinical experience in the field of IPV. Their professional experience spanned from seven years to 28 years. They were asked to evaluate the item pool to assess each item’s relevance to the construct of informal supporter readiness and to ensure comprehensive coverage of all aspects of readiness by the items in the pool. It was decided that unanimous agreement would be required for an item’s inclusion, as recommended for panels of five or fewer judges [43, 44]. Following consultation, each item was unanimously approved as relevant to the construct of informal supporter readiness, thereby affirming the content validity of the ISRI.

Following expert review, proxy target users were invited to assess the items. As the scale is designed to be used by informal supporters within the work of IPV safety advocates, it was determined that professionals within the formal IPV support system, who have direct experience working with survivors, informal supporters, and in developing safety plans, would serve as suitable proxies for the target population review. A total of five IPV survivor support professionals, with roles in child protection, shelters, and outreach advocacy, reviewed the items and provided feedback. The professionals had direct experience working with IPV survivors ranging from five years to 21 years. They were also asked to consider whether any additional questions were needed based on their professional experience. As a result, five additional questions were proposed and unanimously agreed upon for inclusion.

### Scale development

#### Pre-testing questions

Cognitive interviews were conducted with 10 end users across two rounds [38], following the methodology suggested by Beatty and Willis [45]. These interviews employed a combination of the “think aloud” technique and probing questions to assess whether the survey items were easy to understand and interpreted as per the construct definition [46]. Each interview lasted for approximately one hour. Transcripts from these interviews were utilised to identify items that were unclear, signalling the need for revision or removal from the final survey instrument.

#### Survey administration and sample size

The participant sample comprised 357 individuals, 55.5% of whom were female. The mean age was 35.4 years, with a standard deviation of 9.69. Regarding ethnicity, 73.7% identified as Caucasian, 8.6% as Native American, and 7.6% as African American, 5.8% as Latinx, 2.4% as Asian American, and 1.9% as other. In relation to experiences of IPV, 47.2% of the participants reported witnessing frequent or very frequent IPV as a child, while 33.7% reported having experienced IPV as an adult. An additional sample of 50 participants comprising similar demographic composition was recruited to allow for independent reliability testing of the scale. This additional sample comprised 62% females and had a mean age of 33.2 years with a standard deviation of 9.19 years. The ethnic background included 78% Caucasian, 12% African America, and 6% Native American. Of this sample, 42% reported witnessing frequent or very frequent IPV as a child, and 24% reported having experienced IPV as an adult.

#### Procedure

Participants were recruited via the Amazon Mechanical Turk Prime platform (MTurk), where a link to the web-based survey hosted on Qualtrics [47] was advertised. The recruitment period was open from 14/12/2022 to 20/02/2023. Previous research has demonstrated that MTurk samples are efficient and reliable, largely comparable to those obtained through more conventional advertising and recruitment methods [48]. After reading the participant information sheet, participants provided implied consent prior to beginning the survey. Participants first answered demographic questions, including those related to experiences with IPV, and then completed the following scales: the Informal Supporter Readiness Inventory, the Intent to Help Friends Scale [49], and the Generic Job Satisfaction Scale [50]. Each participant received notional compensation through MTurk for the time spent completing the survey.

#### Materials

The measures utilised in this study are detailed below. Table 1 presents the internal reliabilities (Cronbach’s α), theoretical and actual ranges, along with means, and standard deviations for each measure.

**Table 1:** Descriptive statistics for normative factor items.

Sub-Factor	Item	*M*	*SD*
**Subjective Norms**	Believe that although abusive behaviour in a relationship is wrong, you should not interfere as it is a private matter. (R)	5.6	1.0
	Believe that it is important to help and support a domestic violence survivor.	5.6	1.1
	Actively support domestic violence survivors.	5.4	1.1
	Would help someone who was experiencing domestic violence.	5.6	1.1
	Ignore domestic violence as it is a private matter. (R)	5.4	1.2
	Take steps to help domestic violence survivors.	5.6	1.1
**Sense of Belonging**	I have a strong sense of connection with my local community / social network.	5.6	1.1
	It is important to me that I feel a strong sense of connection to my local community / social network.	5.4	1.1
	I feel like I belong in my community / social network.	5.6	1.1
	I am happy that I belong to my social network.	5.5	1.1
	There are times when I feel disconnected from my local community / social network. (R)	5.4	1.1

*Note*. (R) denotes item is reverse coded.

The **Informal Supporter Readiness Inventory** (ISRI) is a newly developed self-report measure designed to gauge the readiness of an individual to support a survivor of IPV. This measure covers three domains: normative, individual, and situational. Participants expressed their level of agreement with each of the 74 statements in the initial item pool on a 7-point scale, where 1 represents "strongly disagree" and 7 represents "strongly agree". An example of an item from the normative factor is: "The important people in my life believe that helping a domestic violence survivor is the right thing to do". An example item from the individual factor is “I know how to support a domestic violence survivor” and an example item from the situational factor is “I have a strong positive relationship with the survivor”. Scores are calculated by summing the responses, with higher scores indicating a greater readiness to provide support. The psychometric properties of this scale are presented in the results section below.

The **Intent to Help Friends Scale** (IHFS; [49]) is a 10-item self-report measure that gauges an individual’s intention to assist or support a known survivor of IPV. Participants rate their likelihood of performing specific behaviours on a five-point scale, where 1 represents "not at all likely" and 5 represents "extremely likely". An example item is: "I would approach someone I know if I thought they were in an abusive relationship and let them know I’m here to help." Scores are calculated by summing the responses and computing an overall average response (between 1 and 5), with higher scores indicating a greater willingness to help or support a DV survivor. The IHFS has demonstrated excellent reliability with a Cronbach’s alpha of .93, and the scale has also shown sound face and construct validity [49]. Cronbach’s alpha was .82 in this sample.

The **Generic Job Satisfaction Scale** (GJS; [50]) is a 10-item self-report measure that assesses an individual’s overall level of satisfaction with their job. Participants express their level of agreement with statements such as "I feel good about my job" on a 5-point scale, where 1 stands for "strongly disagree" and 5 for "strongly agree". Scores are calculated by summing the responses, resulting in a range between 10 and 50. Higher scores indicate a greater overall job satisfaction. The GJS scale has demonstrated acceptable reliability in previous studies with a Cronbach’s alpha of .77 [50]. Cronbach’s alpha was .87 in this sample.

### Statistical analysis

Initially items were removed from the item pool that had problematic wording, or multicollinearity, to ensure the instrument’s clarity, relevance, and appropriateness. The theoretical structure of the scale was then evaluated using confirmatory factor analysis (CFA) using Jamovi [51]. As the CFA approach is theory-driven and designed to confirm a pre-specified model, the rationale for not performing an EFA is justifiable under robust theoretical guidance [52]. As the measure for this study is based on a systematic literature review and pre-existing theoretical model [36], the factor structure is theory driven, rather than inductively derived from the data. It is noteworthy to mention that bypassing an EFA can help mitigate the risk of overfitting to specific sample characteristics, a potential pitfall of EFA, which is primarily data-driven and might capitalise on random variance within a given sample [53]. Multiple fit indices were evaluated, including Chi-square (χ^2^), Comparative Fit Index (CFI), Tucker-Lewis Index (TLI), Root Mean Square Error of Approximation (RMSEA), and Standardised Root Mean Square Residual (SRMR) [54]. The analysis was iterative, with model modifications made based on the results of the CFA and theoretical considerations.

The internal consistency of the factor structure was assessed using Cronbach’s alpha, and a value of Cronbach’s alpha (α > .7) was considered indicative of good internal consistency [55]. Test-retest reliability was evaluated using a Pearson’s product-moment correlation. Convergent validity was evaluated by examining the correlation between the scale and a similar construct (i.e., The Intent to Help Friends Scale; [49]), while divergent validity was assessed by examining the correlation to a distinct construct (i.e., The Generic Job Satisfaction Scale; [50]). A Fisher *r*-to-*z* transformation was used to determine if there was a significant difference between the two correlation coefficients.

## Results

### Scale evaluation

CFAs were employed to test the *a priori* factor structure of the ISRI and its subscales. Before conducting CFA, two items were removed due to multicollinearity and problematic wording. In order to conduct the CFA’s, we recruited a sample size of 357 participants, which exceeded the minimum required convention of 300 participants as suggested by Tabachnick et al. [56].

Separate CFAs were performed for each construct, rather than one larger CFA. The rationale for this approach were threefold. First, conducting individual CFAs provided a more detailed and precise understanding of the structural relationships within each construct, thereby enhancing interpretability [57]. This detailed understanding would aid in more accurately gauging the influence and dynamics of each construct within the larger readiness framework. Second, examining each construct independently allowed for more effective management of potential model fit concerns or multicollinearity. It also offered us the opportunity to implement modifications with greater precision and simplicity [58]. This method increases the reliability and validity of each construct’s measurement, which in turn improves the overall reliability and validity of the ISRI. Third, the practical utility of conducting separate CFAs extends to the potential use of the individual subscales in isolation [59]. Depending on the context, researchers or practitioners may be particularly interested in one of the constructs, and with this approach, each subscale can be used independently while still maintaining its psychometric integrity. Despite necessitating more analyses, the improved interpretability, the enhanced ability to address potential issues, and the flexibility in the practical application of the subscales justified this decision.

#### Original theoretical structure

*Normative factor initial model*. The original theoretical structure comprised of the normative factor, which had three sub-factors consisting of descriptive norms (four items), injunctive norms (four items), and sense of community (six items). Results of the CFA for the initial normative factor model demonstrated the following fit indices: χ^2^(62) = 188, *p* < .001, CFI = .96, TLI = .95, RMSEA = .07, 95% CI [.06, .08], and SRMR = .04. Despite satisfactory CFI and TLI, model fit was considered suboptimal due to the slightly elevated RMSEA [54]. Modification indices suggested the two injunctive items (items 2 and 3) fit better within the descriptive norms subfactor, leaving only one item within the injunctive norms subfactor. Consequently, the injunctive and descriptive norms subfactors were merged into a single factor, termed *’subjective norms*’, aligning with broader literature [60]. Furthermore, item 1 was excluded from subsequent analysis due to its poor factor loading.

*Individual factor initial model*. The initial model for the individual factor which comprised of four subfactors, efficacy (eight items), acceptability of IPV (six items), social responsibility (three items), and experience of violence (four items), displayed a poor fit to the data, with the following indices: χ^2^(714) = 381, *p* < .001, CFI = .89, TLI = .87, RMSEA = .08, 95% CI [.08, .09], and SRMR = .07. In response, item 23 from the efficacy subfactor, due to its poor factor loading, and item 34 from the experience violence subfactor, because of its high residual correlation with item 37 from the experience of violence subfactor, were both removed. Conceptual similarities between the acceptability and social responsibility subfactors led to a reduction in the original theoretical four-factor structure to a three-factor model that maintained alignment with the theoretical definition of the construct.

*Situational factor initial model*. The situational factor was comprised of the following nine subfactors: relationship with the survivor (four items), relationship with the perpetrator (four items), abuse (three items), survivor responsibility (four items), perpetrator responsibility (three items), empathy (five items), risk (four items), readiness (three items), and emotional response (six items). Results of the CFA for the situational factor also demonstrated a poor fit to the data [61], with fit indices: χ^2^(524) = 381, *p* < .001, CFI = .89, TLI = .88, RMSEA = .07, 95% CI = [.06, .07], and SRMR = .08. The initial subpar fit of the model necessitated a more detailed examination of the proposed structure. This led to a theoretical re-evaluation, that considered the sizeable number of subfactors and their theoretical relationships. Our re-evaluation process involved identifying similar elements among the sub-factors and grouping them together based on these shared characteristics. For instance, subfactors relating to emotional and relationship aspects of the situational factor were grouped separately from those pertaining to assessment and responsibility considerations. This resulted in the decision to bifurcate the original situational factor into two distinct factors: one comprising of four subfactors–related to the emotional responses and connections (termed situational-emotion and comprising the survivor relationship, perpetrator relationship, empathy, and emotional response subfactors) and the other encompassing five factors (termed situational-assessment and comprising the survivor responsibility, perpetrator responsibility, risk, abuse, and change readiness subfactors). This revised model was then considered by three of the expert reviewers for theoretical soundness, where the bifurcation was considered to be a more accurate representation of the constructs we were intending to measure. The proposed factors maintained conceptual alignment with the original model while also providing a more fine-grained understanding of the different aspects within the ‘situational’ domain. This new conceptual framework underwent CFA and is presented below.

#### Final factor structure

**Fig 1:**
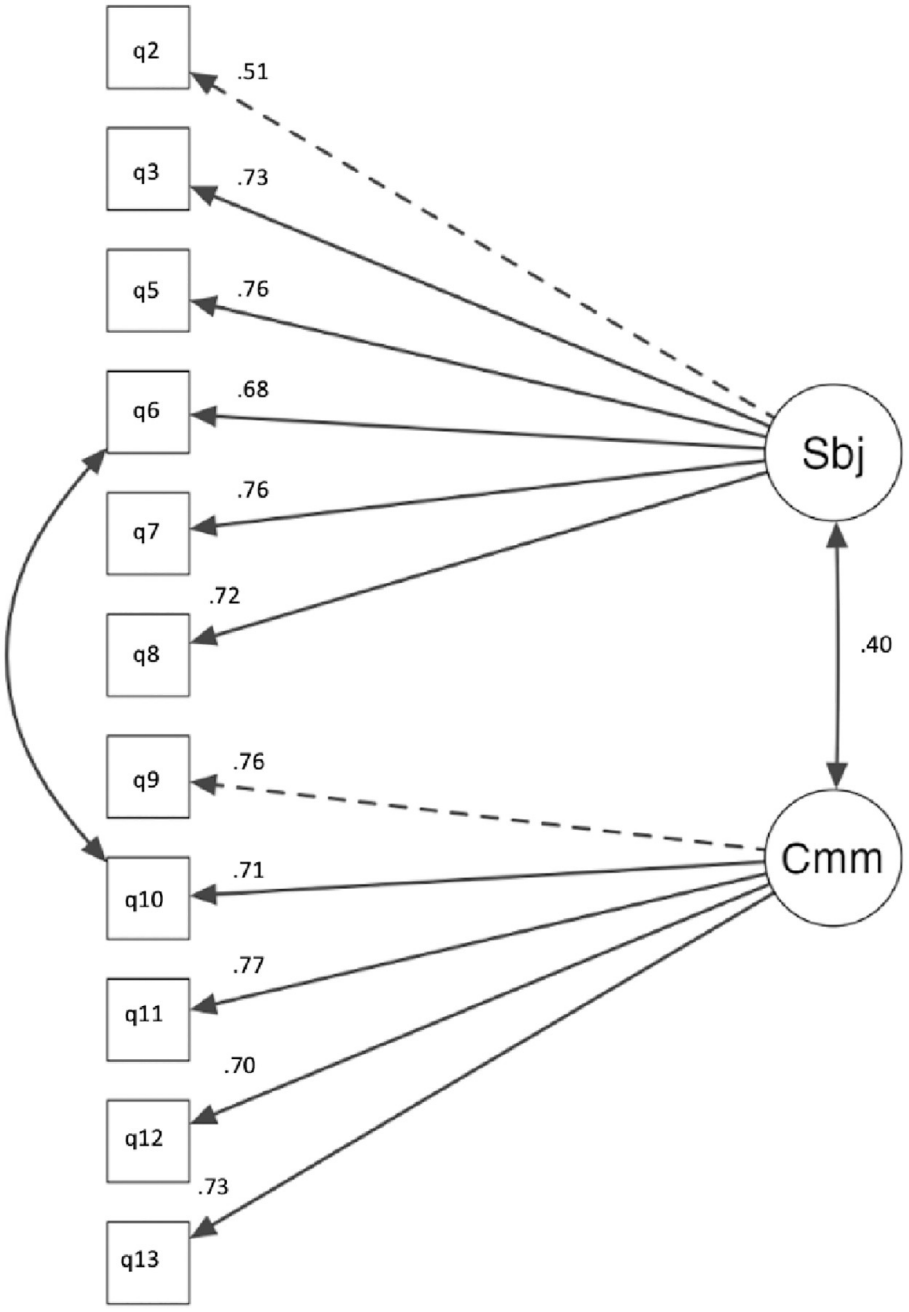
CFA of final normative factor structure. *Note*. The loading for each item is shown above the arrow on the left side. The correlation coefficient for the two sub-factors is shown beside the line between the subfactors.

*Normative factor final model*. The final two-subfactor model for the normative factor consisted of 11 items, see Fig 1. The normative factor items and descriptive statistics are listed in Table 1, with factor loadings and correlations between subfactors displayed in Fig 1. Results of the CFA indicated a strong model fit: χ^2^(42) = 132, *p* < .001, CFI = .96, TLI = .95, SRMR = .04, and RMSEA = .07, 95% CI [.06, .09]. The standardised factor loadings were statistically significant (*p* < .001) and ranged from .51 to .76. The standardised factor loadings for items on the subjective norms (.51 to .76) and sense of community (.70 to .77) subfactors illustrated significant associations with their respective subfactor. The covariance between the subfactors demonstrated a significant, moderate, positive relationship between the subfactors (*r*[355] = .40, *p* < .001) (Akoglu, 2018).

**Fig 2:**
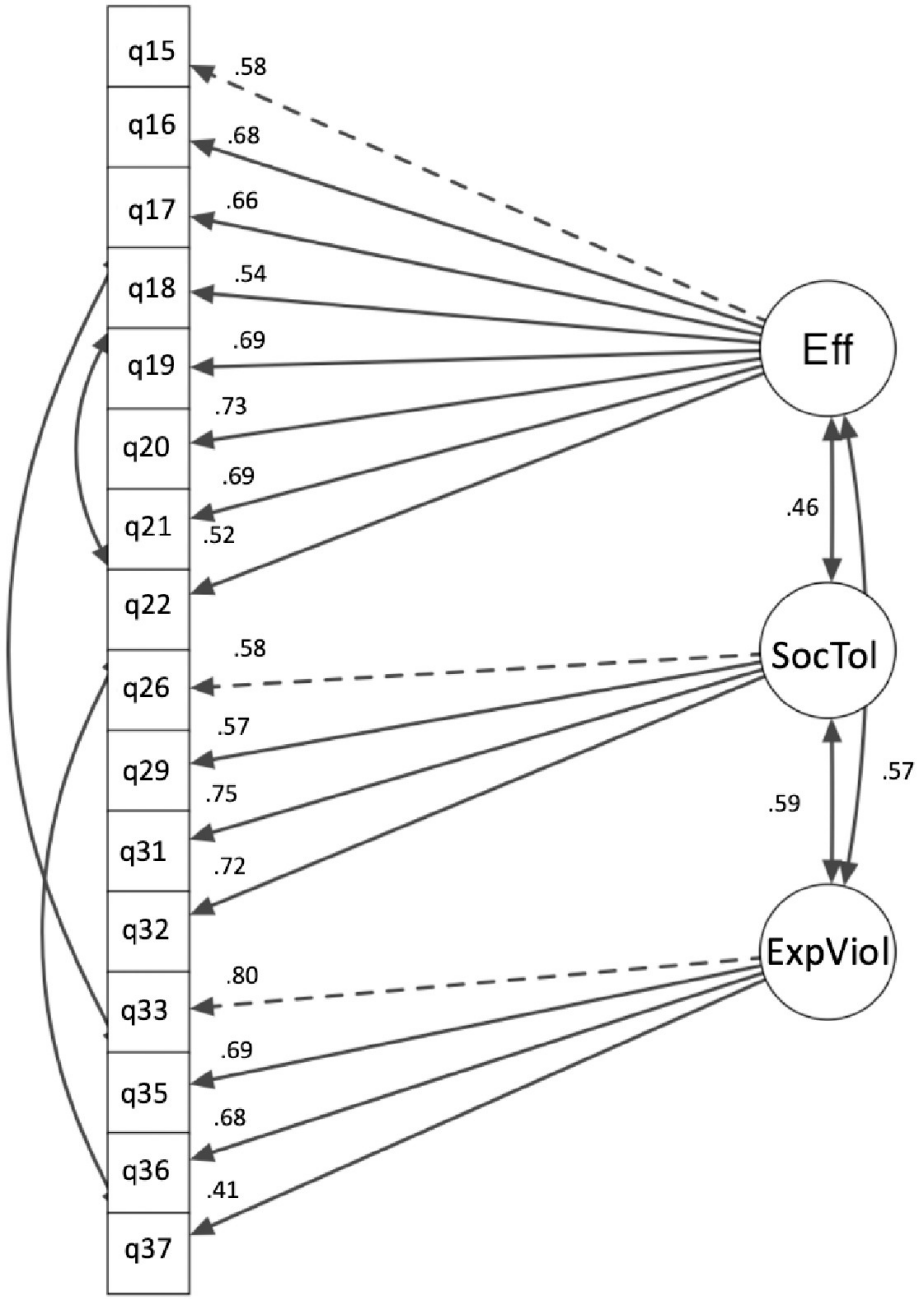
CFA of final individual factor structure. *Note*. The loading for each item is shown above the arrow on the left side. The correlation coefficient for the two sub-factors is shown beside the line between the subfactors.

*Individual factor final model*. The final three-factor model for the individual factor demonstrated an acceptable fit to the data: χ^2^(97) = 328, *p* < .001, CFI = .92, TLI = .91, SRMR = .05, RMSEA = .80, 95% CI [.07, .09]. All standardised factor loadings, factor covariances, and residual covariances were statistically significant (*p* < .001), highlighting that the observed variables were significantly associated with their respective latent factors. The scale items and descriptive statistics for the individual factor are listed in Table 2. The factor loadings and correlations between factors are displayed in Fig 2.

**Table 2:** Items and descriptive statistics for the individual factor.

Sub-Factor	Items	*M*	*SD*
**Efficacy**	I know how to support a domestic violence survivor.	5.5	1.1
	I would know what to do if I found out someone close to me was experiencing domestic violence in their relationship.	5.4	1.2
	If I suspected someone close to me was experiencing domestic violence, I would feel confident to talk to them about it.	5.4	1.2
	It would be difficult to start a conversation about domestic violence with a survivor who was close to me. (R)	5.3	1.3
	I am able to support a survivor to access professional services they might need.	5.4	1.2
	I know where to go to find community supports available to survivors.	5.5	1.2
	I would provide support to a domestic violence survivor who was close to me.	5.5	1.2
	I would find it difficult to provide support to someone close to me if they were experiencing domestic violence. (R)	5.2	1.4
**Social Tolerance**	There are no situations where a man should be abusive towards his partner.	5.5	1.2
	Sometimes men must use acts of domestic violence to keep their relationship in order. (R)	5.4	1.4
	I believe I have a role in stopping domestic violence.	5.5	1.2
	As a member of society, I have a role in ending domestic violence.	5.6	1.1
**Exposure to Violence**	*I believe my own experiences of violence*:		
	Have prepared me to be able to support a domestic violence survivor.	5.5	1.1
	Give me insight into how to best support a domestic violence survivor.	5.4	1.2
	Make me a better informal supporter.	5.5	1.2
	Would leave me triggered if I supported a domestic violence survivor. (R)	5.1	1.5

*Note*. (R) denotes item is reverse coded.

**Fig 3:**
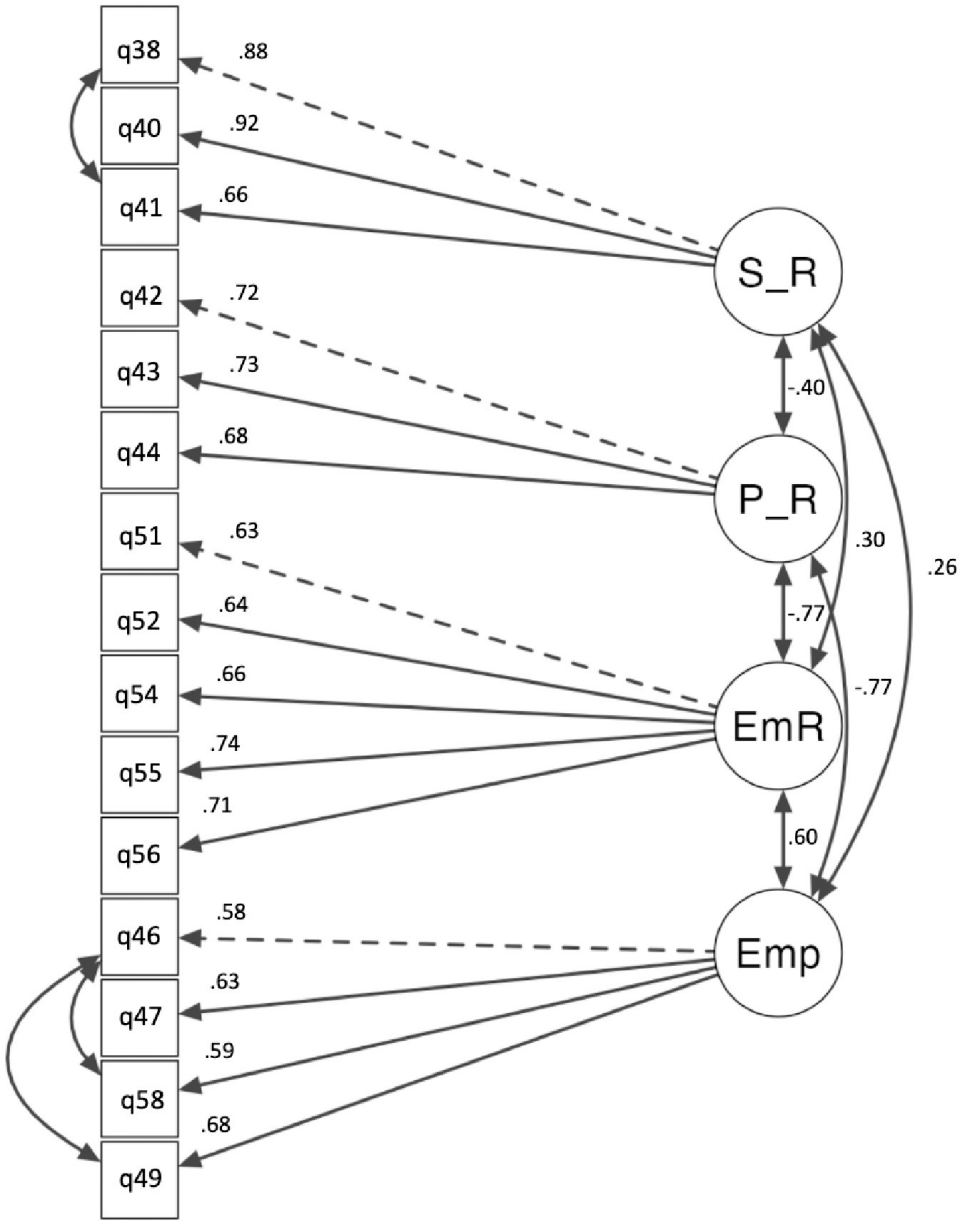
CFA of final situational-emotional response factor structure. *Note*. The loading for each item is shown above the arrow on the left side. The correlation coefficient for the four sub-factors is shown beside the line between the subfactors.

*Situational factor final model*. As discussed above, the situational factor was separated into two distinct factors. The first being situational-emotional, which was examined across four factors: survivor relationship, perpetrator relationship, emotional response, and empathy. The model indicated a satisfactory fit: χ^2^(81) = 242, *p* < .001, CFI = .94, TLI = .92, RMSEA = .08, 95% CI [.07, .09], SRMR = .06. All standardized factor loadings, factor covariances, and residual covariances were statistically significant (*p* < .001), highlighting that the observed variables were significantly associated with their respective latent factors. The scale items and descriptive statistics for the situational-emotional factor are listed in Table 3. The factor loadings and correlations between factors are displayed in Fig 3.

**Table 3:** Items and descriptive statistics for the situation-emotional factor.

Sub-Factor	Items	*M*	*SD*
**Relationship -Survivor**	I have a strong positive relationship with the survivor.	5.4	1.1
	There is often conflict in my relationship with the survivor. (R)	5.3	1.0
	I could count on the survivor to help me if I had a problem.	5.4	1.3
**Relationship -Perpetrator**	I have a strong positive relationship with the perpetrator. (R)	4.3	1.5
	The perpetrator is an important person in my life. (R)	4.1	1.5
	There is often conflict in my relationship with the perpetrator.	4.2	1.4
**Emotional Response**	I have hope that supporting the survivor will make things better.	5.4	1.3
	I would feel guilty if I ignored the survivor.	5.3	1.4
	Learning that the survivor was experiencing domestic violence made me feel very angry. (R)	5.3	1.4
	Thinking about the survivor’s experience of domestic violence makes me feel anxious. (R)	5.3	1.3
	When I think about the survivor’s experience I am overcome with emotions. (R)	5.4	1.2
**Empathy**	I felt empathy for the survivor.	5.4	1.3
	I could understand what the survivor was going through.	5.4	1.3
	I found it difficult to know what goes on in the mind of the survivor. (R)	5.2	1.4
	I can imagine how the survivor felt.	5.4	1.3

*Note*. (R) denotes item is reverse coded.

**Fig 4:**
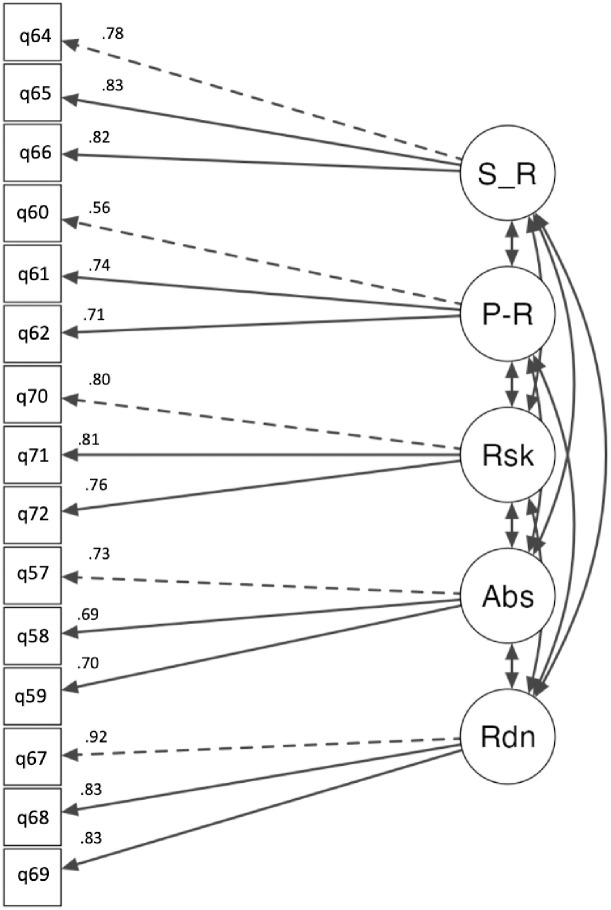
CFA of final situational-assessment factor structure. *Note*. The loading for each item is shown above the arrow on the left side.

The additional factor named situational-assessment was examined across five sub-factors. The sub-factors analysed included survivor responsibility, perpetrator responsibility, risk, abuse, and change readiness. The model fit indices suggested a good fit: χ^2^(80) = 154, *p* < .001, CFI = .97, TLI = .97, SRMR = .03, and RMSEA = .05, 95% CI [.04, .06]. The standardised factor loadings were statistically significant (*p* < .001) and ranged from .558 to .924. The standardised factor loadings for each factor—survivor responsibility (.78 to .83), perpetrator responsibility (.71 to .74), risk (.76 to .81), abuse (.69 to .73), and change readiness (.82 to .83)—all illustrated significant associations with their respective factors. The factor covariances demonstrated notable relationships between the factors, with standardised estimates ranging from -.46 to .98 (*p* < .001). All factor inter-correlations were statistically significant at *p* < .001. The residual covariances ranged from .31 to .69, and the residual intercepts varied between 2.9 and 5.5. The residual estimates were also statistically significant (*p* < .001). The scale items and descriptive statistics for the situational-assessment factor are listed in Table 4. The factor loadings and correlations between factors are displayed in Fig 4.

**Table 4:** Items and descriptive statistics for the situational-assessment factor.

Sub-Factor	Items	*M*	*SD*
**Responsibility–Survivor**	The survivor provoked her partners abusive behaviour. (R)	5.4	1.2
	I felt that the survivor brought the experience on herself. (R)	5.3	1.3
	The survivor’s actions caused the domestic violence. (R)	5.3	1.3
**Responsibility–Perpetrator**	I think the perpetrator was responsible for his choice to be abusive.	4.1	1.5
	I think the perpetrator was in control of his actions.	4.2	1.5
	I think the perpetrator decided to use acts of domestic violence.	4.2	1.4
**Risk**	Worrying about my own safety was a big factor in deciding whether or not to support the survivor.	5.1	1.5
	Worrying about the safety of my family was a big factor in deciding whether or not to support the survivor.	5.1	1.5
	Worrying about the safety of the survivor if her partner knew I was supporting her was a big factor in deciding whether or not to provide support.	5.1	1.4
**Change Readiness**	I felt that the survivor was not ’ready’ for change or to receive support. (R)	5.3	0.9
	It was the right time to provide support to the survivor.	5.4	1.2
	I knew the survivor was in the ’right place’ to receive support.	5.4	1.1
**Abuse**	*In my opinion*:		
	the survivor was experiencing severe domestic violence.	5.5	1.2
	the survivor was experiencing frequent domestic violence.	5.3	1.2
	the survivor was experiencing ongoing domestic violence.	5.3	1.3

*Note*. (R) denotes item is reverse coded.

### Tests of reliability

The internal consistency of the four factors of the ISRI (normative, individual, situational-emotional, and situational-assessment) were assessed using Cronbach’s alpha with the original sample, Sample 1, which consisted of the 357 participants involved in the CFA. Additionally, to replicate reliability and affirm measurement equivalence across diverse samples [62], a second independent sample, Sample 2, comprising 50 participants, was employed. In Sample 1, the Cronbach’s alpha values for the normative, individual, situational-emotional, and situational-assessment subscales were found to be .92, .89, .85, and .88, respectively. In sample 2, the corresponding Cronbach’s alpha values were .89, .87, .89, and .84. Collectively, these results indicate that the four factors of the ISRI exhibit good to excellent internal consistency across both samples [55].

The test-retest reliability of the ISRI was assessed using Pearson’s correlations. Streiner et al. [63] noted that the ideal duration between assessments can change, influenced by factors such as the attribute under examination, the consistency of the attribute over time, and the population under study. Therefore, as other scales quantifying aspects of IPV have used time points between four and eight weeks [64, 65] we opted for a six-week retest period. A total of 63 participants from sample 1 completed the measure at the two timepoints. All four factors had excellent test-retest reliability. Mean scores and correlations between the timepoints are displayed in Table 5.

**Table 5:** Test-retest reliability of the ISRI factors.

Factor	Timepoint 1	Timepoint 2	*r*	95% CI Lower	95% CI Upper	Sig
	*M* (*SD*)	*M* (*SD*)				
Normative	58.6 (6.3)	57.2 (7.3)	.78	.67	.86	< .001
Individual	84.3 (9.5)	83.3 (10.7)	.85	.76	.91	< .001
Situational-Emotion	80.7 (17.6)	81.3 (14.8)	.94	.90	.96	< .001
Situational-Assessment	59.6 (13.6)	75.9 (14.4)	.77	.66	.86	< .001

### Tests of validity

To investigate hypothesis 3, exploring convergent validity, the correlation between the ISRI scale and the Intent to Help Friends Scale [49] was calculated. Results indicated a strong positive correlation (*r*[355] = .73, *p* < .001) [66], suggesting that the ISRI is measuring a construct similar to the one captured by the Intent to Help Friends Scale, thereby supporting convergent validity. Additionally, divergent validity was examined by correlating the ISRI with the Generic Job Satisfaction scale [50], and the correlation coefficient between these two scales was moderate (*r*[355] = .34, *p* < .001) [66], suggesting that the ISRI is measuring a construct distinct from job satisfaction, thereby providing support for divergent validity. Finally, a Fisher *r*-to-*z* transformation was computed to determine whether these correlations were significantly different from each other. The results were significant (*z* = 8.34, *p* < .001), which suggests that there is a significant difference between the ISRI’s strong correlation with the Intent to Help Friends Scale and its moderate correlation with the Job Satisfaction Scale. This finding supports our assertion that the ISRI has sound convergent and divergent validity.

**Fig 5:**
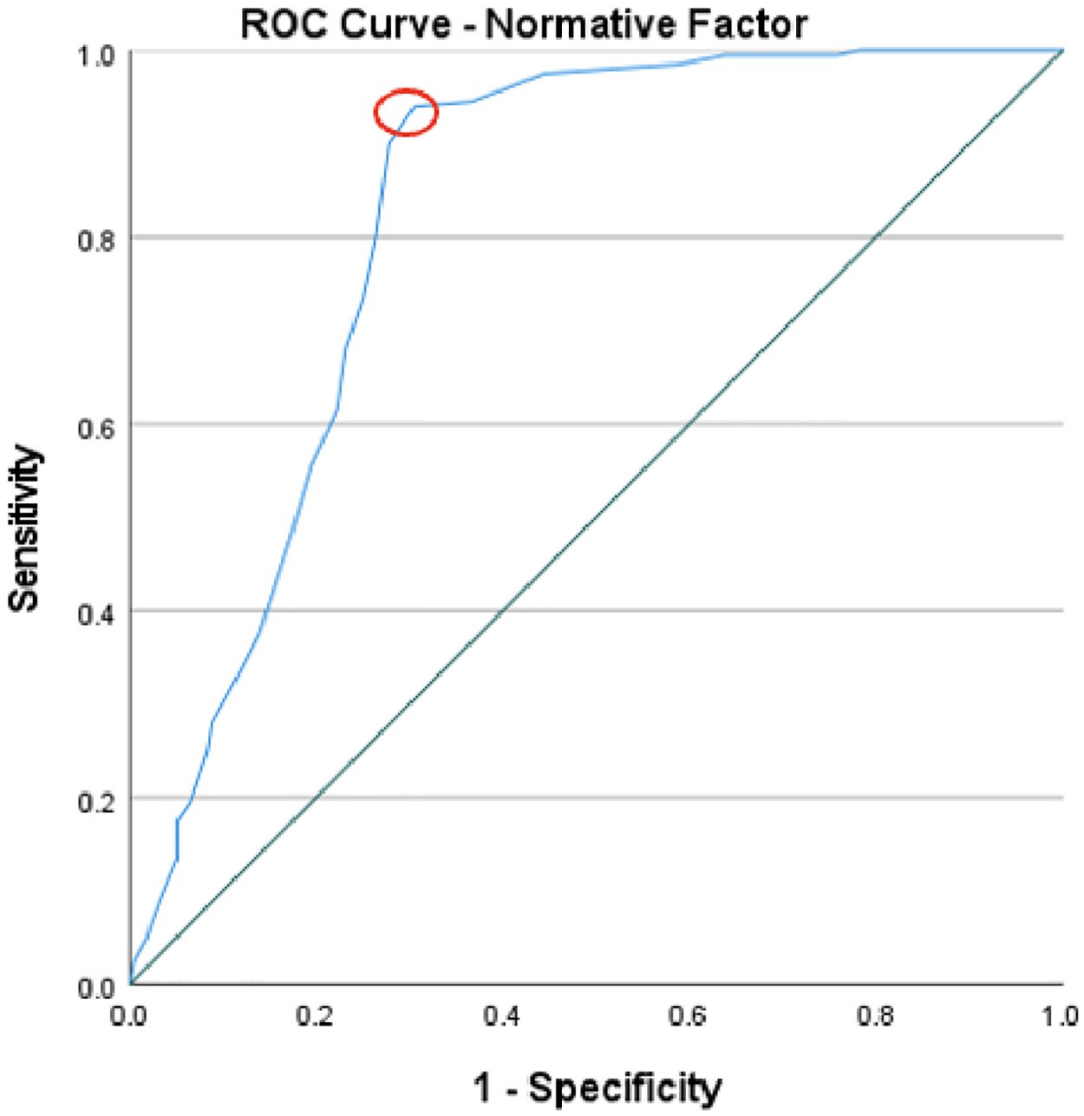
ROC curve of the normative factor’s predictive value for informal supporter readiness.

**Fig 6:**
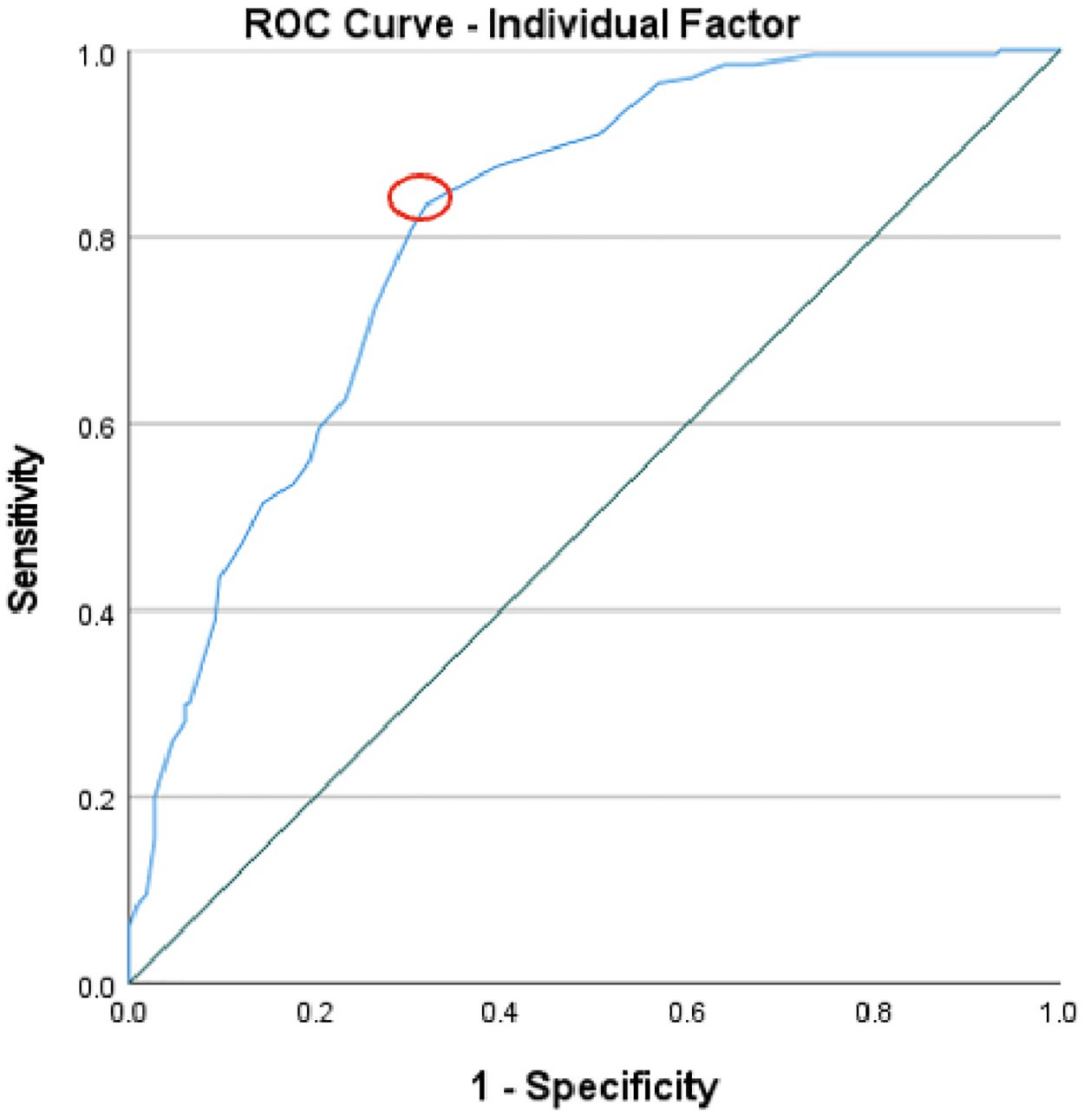
ROC curve of the individual factor’s predictive value for informal supporter readiness.

**Fig 7:**
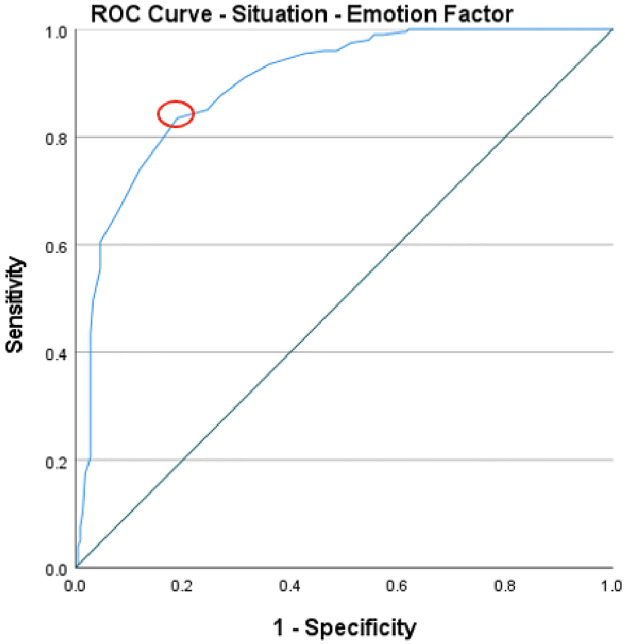
ROC curve of the situational-emotion factor’s predictive value for informal supporter readiness.

**Fig 8:**
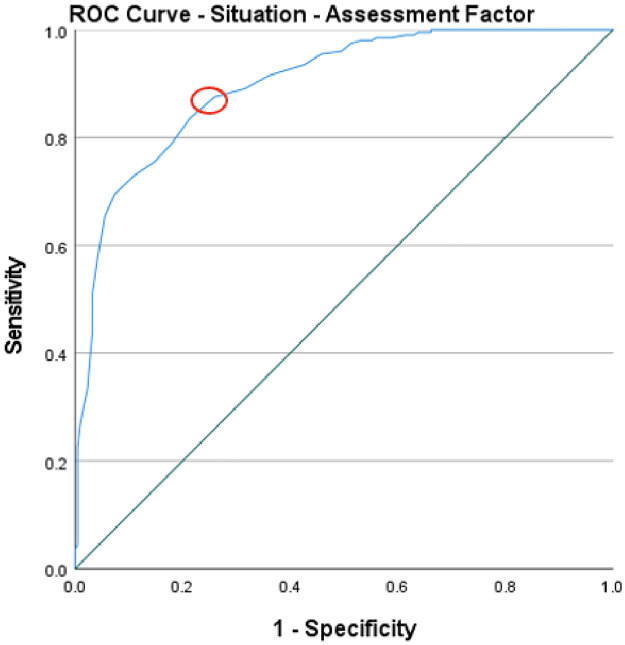
ROC curve of the situational-assessment factor’s predictive value for informal supporter readiness.

To evaluate the predictive validity of the ISRI, Receiver Operating Characteristics (ROC) curve analysis was used across the four factors. The normative factor, with an area under the curve (AUC) of .82, illustrated a good level of predictive accuracy and suggests that there is an 82% chance that a randomly picked positive instance (high readiness) would rank higher than a negative one (low readiness). The optimal threshold, based on Youden’s index which maximises the sum of sensitivity and specificity, was found to be 60 (Youden’s index .62), which could be used to distinguish between instances of high and low readiness. Fig 5 displays the cut-off point, indicated with a red circle, corresponding to the “point on the ROC curve with the highest vertical distance from the 45° diagonal line” [67]. The individual factor had an AUC of .81, implying a good level of predictive accuracy, with an 81% chance of a random positive instance exhibiting a higher level of help-giving readiness than a negative one. A cut-off value of 85 (Youden’s index .52) provides a benchmark for classification. Fig 6 displays the cut-off point for the individual factor. When evaluating the situational-emotional factor, an AUC of .90 was identified, signifying an excellent level of predictive accuracy, and a 90% probability of a positive instance scoring higher than a negative instance. The cut-off of 71 (Youden’s index .65) separates the instances into high and low readiness levels. Fig 7 depicts the cut-off point for this factor. Lastly, the situational-assessment factor revealed an AUC of .90, denoting an excellent level of predictive accuracy, and a 90% chance that a randomly picked positive instance would rank higher than a negative one. An optimal threshold of 75 (Youden’s index .62) was identified to classify the instances into low and high readiness. As with the previous factors, Fig 8 displays the cut-off point.

**Fig 9:**
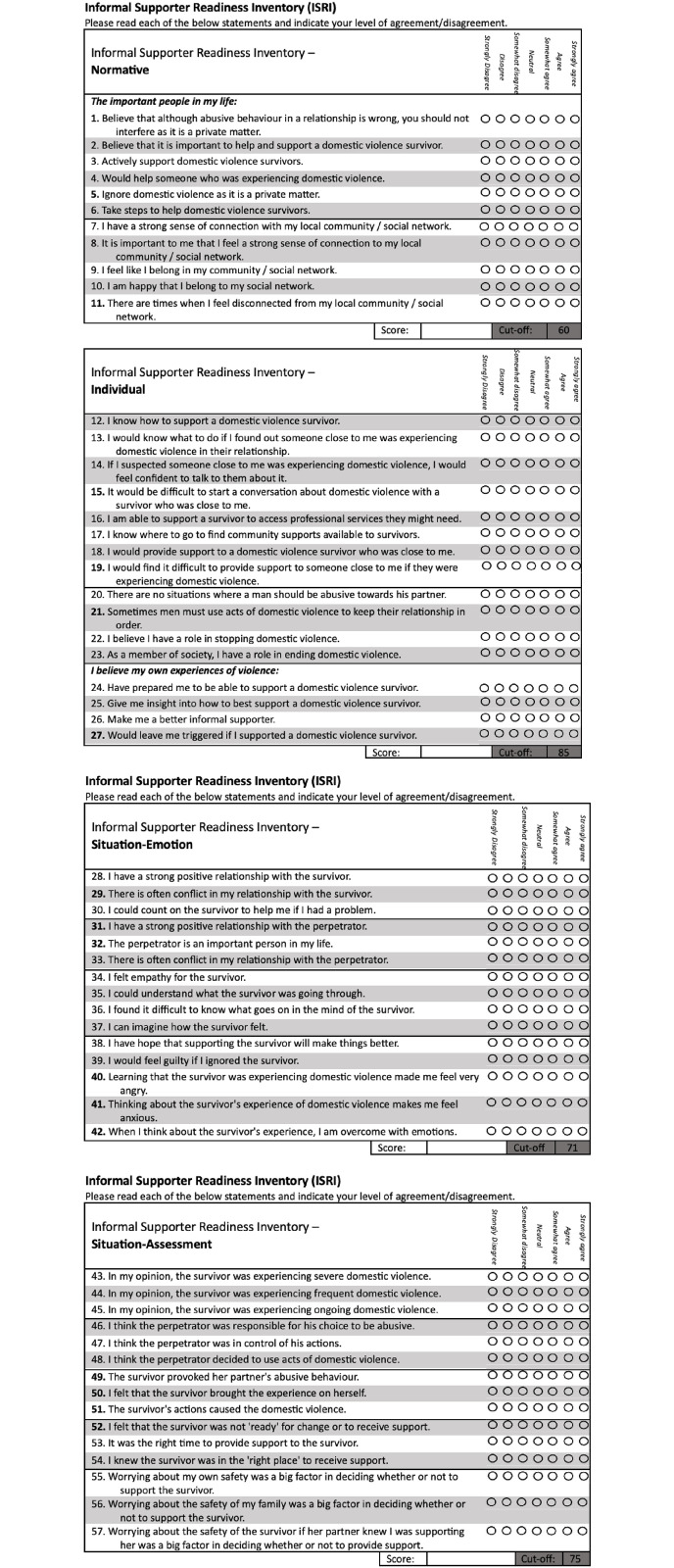
Final items and response format for ISRI.

**Fig 10:**
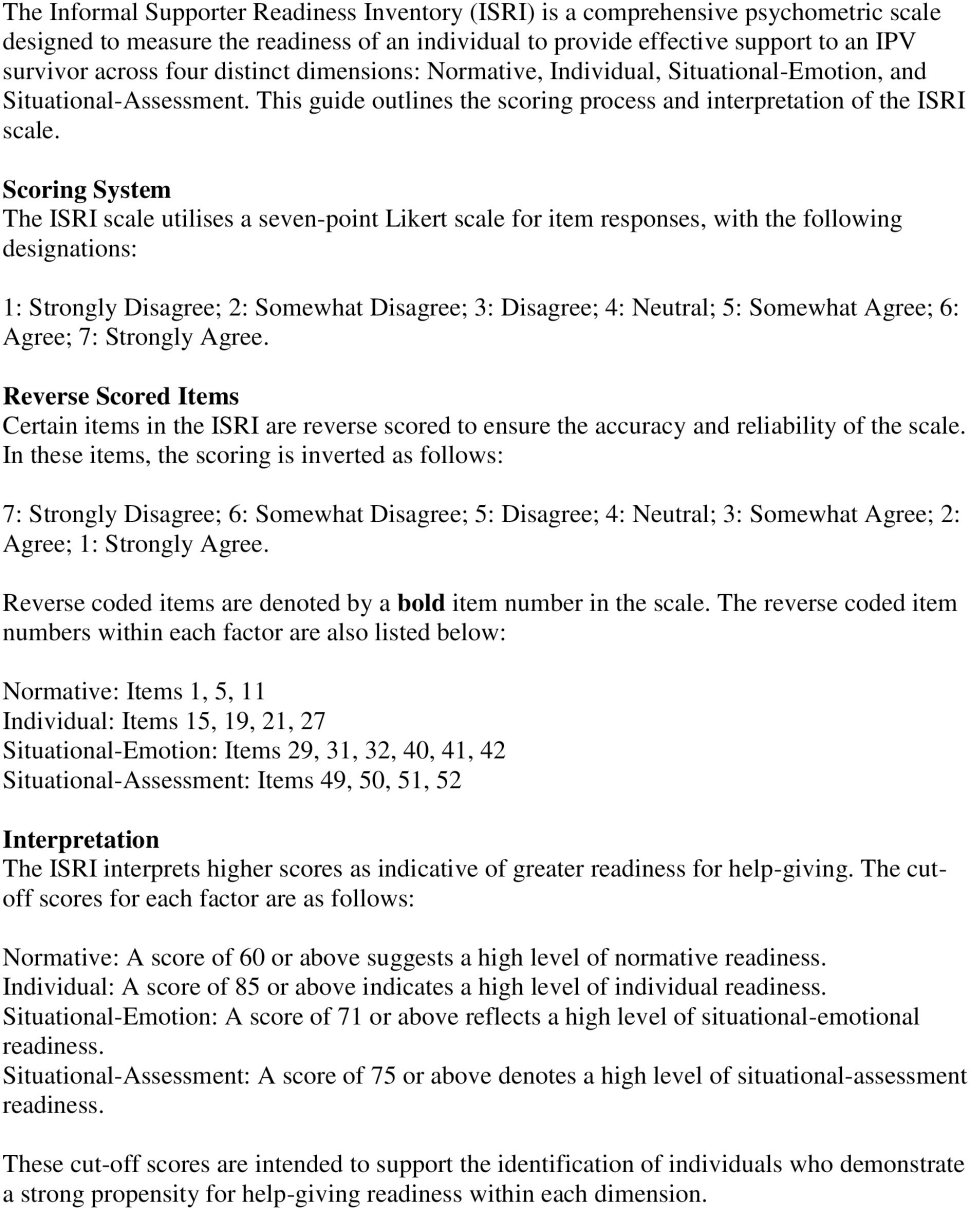
Scoring and interpretation instructions for the ISRI.

The final items of ISRI are presented in Fig 9. This figure offers an overview of each ISRI factor, delineating the specific items that contribute to each construct, their response format, and the associated cut-off scores that indicate clinical significance. Fig 10 provides additional guidance on scoring and interpterion.

## Discussion

The aim of this research was to the develop and validate a quantitative measure of the factors that inform social network members’ responses to IPV situations–the Informal Supporter Readiness Inventory (ISRI). Following the best practice guidelines specified by Boateng et al. [38], this research found support for the elements of the Model of Informal Supporter Readiness [36]. Based on the literature, this model consists of three broad primary factors (normative, individual, and situational) that are related to the intervention of social network members. Confirmatory Factor Analyses supported the factor structure of the normative and individual factors and bifurcated the situational factor into two discrete factors of situational-emotional and situational-assessment. The four new factors exhibited robust internal consistency. The ISRI and its components also demonstrated excellent test-retest reliability and strong support for convergent, divergent, and predictive validity providing initial empirical support for the ISRI as a valid and reliable tool, capable of offering unique insights into understanding social network member interventions and the multifaceted nature of support in IPV contexts. Each of the four factors comprises distinct sub-factors which offer further insight into the intricate nature of informal supporter helping behaviour, and showcase the complex interplay of societal norms, individual values, emotional reactions, and risk assessments in shaping helping readiness.

In particular, the normative factor, encapsulating societal and cultural norms surrounding help-giving behaviour, was found to be correlated with help-giving readiness, emphasising the pervasive role of social norms in predicting helping readiness. Similarly, the individual factor, containing personal values and attitudes, supported that personal attributes are correlated to readiness. The situational-emotional and situational-assessment factors, together, furthered current conceptions of help-giving behaviour by revealing the complex dynamics of relationships with both the perpetrator and the survivor, and the role they may play in intervention decisions, which has not previously been considered. It was noted that an informal supporter’s emotional reactions to the survivor and their evaluations of the risk and severity of the situation are associated with their decision to intervene.

Although our initial theoretical framework served as a strong guide, we identified some areas that required refinement. For instance, we found that situational factors [36] were more accurately represented by two distinct factors of situational-emotional and situational-assessment. This separation provides a more detailed understanding of how emotional responses and rational evaluations may independently shape informal supporter intervention decisions. This conceptualisation provides a more granular insight into the varying situational elements that informal supporters may consider, enhancing our understanding of the complexity involved in decision-making processes during IPV incidents. This refined perspective may improve the design of intervention programs by addressing these distinct situational elements, ultimately driving more effective and individualised support strategies for those involved in such scenarios.

### Comparison with previous measures

The development of the ISRI provides progression in informal supporter research within IPV contexts and promotes the further integration of social network-oriented approaches to survivor safety. The ISRI is novel compared to previous measures, such as those that have primarily focused on informal supporter ’willingness’ [35], by delving deeper to explore factors other than intention which are associated with actual helping behaviour [36]. This shift from behavioural intention to actual behaviour provides a more comprehensive representation of informal support. Moreover, the ISRI includes the dynamic of relationships with both the survivor and the perpetrator within situational factors, a perspective absent in previous measures. This added consideration enhances understanding of critical dynamics that may influence intervention decisions. By integrating current literature on normative and individual factors, the ISRI not only provides a multidimensional framework for understanding informal supporter intervention but also establishes itself as a tool that emphasises the importance of context and actual behaviour in intervention decisions.

### Implications

Subject to successful trialling and evaluation, the implementation of the ISRI in practice could considerably enhance the efficacy of interventions in IPV situations. This measure may afford a comprehensive understanding of informal supporters’ readiness to help, accounting for factors including their attitudes, belief systems, emotional responses, and assessments of situational elements. This detailed insight may facilitate the tailoring of supportive interventions, allowing for more targeted and individualised strategies to enhance readiness and support capacities.

The practical benefits of using the ISRI are augmented by a shift towards a social network-oriented approach [13, 30]. In this approach, formal services, including IPV safety advocates, counsellors, and shelter workers, would actively collaborate with the survivor’s social network to build a more robust support system. This approach recognises and leverages the crucial role of informal supporters, integrating them more effectively into the broader network of assistance.

Importantly, the readiness assessment provided by the ISRI can inform the development of the survivor’s safety plan. By identifying who in their network is most prepared to provide support, survivors can better decide who to include in their safety plan, fostering a network of support that is not only willing but also capable. Therefore, the ISRI in promoting this social network-oriented approach, could play a crucial role in strengthening the web of support surrounding survivors, thereby enhancing their safety and well-being.

From a research perspective, the introduction of the ISRI holds potential for meaningful contributions to IPV research. By shifting focus from the bystander model’s concept of ’willingness’ to a more comprehensive notion of ’readiness’, the ISRI can offer a nuanced understanding of IPV situations. This could allow researchers to delve deeper into the multifaceted factors influencing a supporter’s ability to provide sustained assistance, thereby extending our theoretical understanding of IPV situations. By potentially moving beyond the limitations of previous models, the ISRI may provide researchers with a more robust and detailed lens through which to assess the role of support within IPV situations.

### Future research directions

Future research is needed to validate the ISRI across a broader range of contexts and samples. This includes focusing on diverse environments and demographic groups, such as cultural minorities, to assess the measure’s efficacy across different populations. It would also be beneficial to explore how different demographic variables, such as age, gender, and socio-economic status, might correlate with readiness scores. Additionally, longitudinal studies examining the stability of readiness over time could further explain the dynamic nature of informal supporter readiness and its relationship with actual helping behaviour, particularly in situations of extended or chronic IPV.

### Limitations

Despite the informative findings this study presents, certain limitations must be acknowledged. Foremost, the potential for self-report bias is present, as the participants may have overestimated their readiness as informal supporters.

Moreover, the sample was predominantly female, which could raise concerns about gender bias. However, it is important to highlight that in real-world contexts, particularly those involving tertiary interventions where ongoing support is provided, most informal supporters are women [14]. Hence, our study might provide a representative portrayal of the typical informal supporter’s profile. Nevertheless, further research should aim for more gender-balanced samples to better assess diverse supporter experiences and readiness levels.

In terms of the MISR [36], there were some departures identified by the CFAs, notably the bifurcation of situational factors into situational-emotional and situational-assessment. These discrepancies, however, enrich our understanding of the complex and multifaceted nature of readiness in informal supporters, prompting the need for continuous refinements in our theoretical understanding.

## Conclusion

In conclusion, this study presents an advancement in understanding the readiness of informal supporters in assisting survivors of IPV. By developing and validating the ISRI, we contribute a tool which enables quantifiable and in-depth insights into informal supporters’ readiness to be gathered. The key findings align closely with our MISR [36], yet also reveal complexities like the bifurcation of situational factors. Practical and research applications are multifaceted, from strengthening support networks and empowering survivors in making informed decisions about safety planning, to supporting the future direction of IPV research by facilitating a more comprehensive exploration of survivor support systems. This research highlights the benefits of adopting a social network-oriented approach and fostering collaboration between formal services and informal supporters. We anticipate that these contributions will prompt further research and practical advancements, thereby enhancing the support provided to IPV survivors.
